# Barriers to Video Call–Based Telehealth in Allied Health Professions and Nursing: Scoping Review and Mapping Process

**DOI:** 10.2196/46715

**Published:** 2023-08-01

**Authors:** Lena Rettinger, Sebastian Kuhn

**Affiliations:** 1 Health Assisting Engineering FH Campus Wien, University of Applied Sciences Vienna Austria; 2 Institute of Digital Medicine University Hospital of Giessen and Marburg Philipps University Marburg Marburg Germany

**Keywords:** telehealth, telemedicine, eHealth, barriers, allied health professions, nursing, video call, videoconferencing, web-based consultation, remote consultation, mobile phone

## Abstract

**Background:**

Telehealth interventions have become increasingly important in health care provision, particularly during the COVID-19 pandemic. Video calls have emerged as a popular and effective method for delivering telehealth services; however, barriers limit the adoption among allied health professionals and nurses.

**Objective:**

This review aimed to identify and map the perceived barriers to the use of video call–based telehealth interventions among allied health professionals and nurses.

**Methods:**

A comprehensive literature search was conducted in the PubMed and CINAHL databases on June 22, 2022, and updated on January 3, 2023, following the PRISMA-ScR (Preferred Reporting Items for Systematic Reviews and Meta-Analyses extension for Scoping Reviews) guidelines. Only original studies published in English or German since June 2017 that reported barriers to the use of video call–based telehealth interventions were eligible for inclusion. The studies had to involve interviews, focus groups, or questionnaires with physical therapists, occupational therapists, speech and language therapists, audiologists, orthoptists, dieticians, midwives, or nurses. Each publication was coded for basic characteristics, including country, health profession, and target group. Inductive coding was used to identify the patterns, themes, and categories in the data. Individual codings were analyzed and summarized narratively, with similarities and differences in barriers identified across health professions and target groups.

**Results:**

A total of 56 publications were included in the review, with barriers identified and categorized into 8 main categories and 23 subcategories. The studies were conducted in various countries, predominantly the United States, Australia, the United Kingdom, Canada, Israel, and India. Questionnaires were the most commonly used evaluation method, with 10,245 health professionals involved. Interviews or focus groups were conducted with 288 health professionals. Most of the included publications focused on specific health care professions, with the highest number addressing barriers for physical therapists, speech and language therapists, and audiologists. The barriers were related to technology issues, practice issues, patient issues, environmental issues, attributions, interpersonal issues, policies and regulations, and administration issues. The most reported barriers included the lack of hands-on experience, unreliable network connection, the lack of technology access, diminished fidelity of observations and poor conditions for visual instructions, the lack of technology skills, and diminished client-practitioner interaction and communication.

**Conclusions:**

This review identified key barriers to video call–based telehealth use by allied health professionals and nurses, which can foster the development of stable infrastructure, education, training, guidelines, policies, and support systems to improve telehealth services. Further research is necessary to identify potential solutions to the identified barriers.

## Introduction

### Background

Allied health professionals and nurses are an integral part of our health care system. The allied health professions do not constitute a clearly defined group of professions, as definitions and classifications vary at the international level [1]. For the purposes of this review, the allied health professions are occupational therapists (OTs), physical therapists (PTs), speech and language therapists (SLTs), audiologists, dietitians, orthoptists, and midwives. In addition, nurses were identified as a crucial professional group within the context of allied health services. Allied health professionals and nurses have in common that they play an important role in patient care to protect, restore, and maintain physical, sensory, psychological, cognitive, social, and cultural functions [2].

The COVID-19 pandemic has profoundly challenged the work of health care professionals because of contact restrictions and the risk of infections [3,4]. Access to health services was limited worldwide [5,6] and affected, among others, access to rehabilitation services [7,8] and maternal health services [9]. The health care contacts of acute [10] and chronically ill patients [10-12] decreased during this period. Therefore, the pandemic has led to fundamental changes in service provision and advanced the integration of telehealth services in various disciplines and health fields [13]. Telehealth offers advantages for allied health professionals by overcoming barriers related to distance [14] and addressing health and services disparities [15-17]. This facilitates improved access to services and supports individuals with chronic illnesses in maintaining continuity of care, ultimately optimizing health and well-being [15,16,18]. However, there remains a concern that telehealth may inadvertently exacerbate health care disparities for susceptible populations, particularly those with limited digital literacy or restricted access to digital resources [19].

Telemedicine or telehealth can be defined as “the use of information and communication technology (ICT) to provide health services where there is physical separation between providers of care and/or recipients over long and short distances” [20]. Terminology in this area lacks agreement on the definitions of the telehealth or telemedicine concepts [21]. Telehealth might be related to telemedicine in the same way that health is related to medicine [22]. Consequently, the World Medicine Association describes telehealth and telemedicine to be used for remote clinical services to serve for patient-physician consultation where access is limited and to be used for consultation with ≥2 physicians [23]. Furthermore, telehealth refers to remote clinical and nonclinical services, such as preventive health support, research, training, and continuing medical education for health professionals [23]. Similar terms, such as telerehabilitation, telecare, telepractice, or telephysiotherapy, refer to specializations, the context of care, or a specific profession. Telehealth services can be divided into 3 types: synchronous, where health information is delivered in real time; asynchronous, where health information is stored and forwarded; and patient monitoring, where patient clinical status is continuously evaluated over the distance [24].

Video calls are a common method for providing telehealth services synchronously, as they have become prevalent in personal and professional lives. Everyday technologies, such as computers, tablets, smartphones, webcams, videoconferencing software, and an internet connection, make such services relatively accessible. Especially during the COVID-19 pandemic, there was a demand for readily available solutions without the need for additional infrastructure or equipment. The rapid implementation of video visits with health care professionals prevented infections [25] and kept the health care system running [26-29]. For instance, telehealth services offered by physiotherapists were used to support patients in isolation [30] or to address the ongoing needs of children with neurodevelopmental or musculoskeletal issues [31]. This approach enabled the provision of acute care while maintaining continuity in therapeutic interventions.

Telehealth also has potential benefits in addition to the prevention of infections. Such services have been established long before facing a pandemic, especially in rural areas, to increase access to physical or occupational therapy, speech and language therapy, nursing, and other health services [32-34]. People who are bedridden or have limited mobility can also benefit from easier access [35]. Furthermore, increased independence and reassurance have been described through telehealth use [36]. Studies have shown that telehealth can yield equivalent or even superior clinical outcomes for patients [18,37,38]. However, such services are not a common component of health services.

### Objectives

The implementation of telehealth faces organizational, personal, and technological barriers, such as the lack of infrastructure, missing skills, poor strategic alignment, resistance to necessary cultural changes, and cost or reimbursement issues [39]. However, to date, no review has systematically mapped these barriers. An illustration and analysis of telehealth barriers is important to address them in the future by tailored actions such as training, organizational redesigns, or infrastructure measurements.

To systematically map barriers to video call–based telehealth from nonmedical health professionals, a mapping and scoping review with the following research question was conducted: What barriers are indicated and described by allied health professionals and nurses toward video call–based telehealth?

## Methods

### Overview

First, a scoping review was conducted based on the PRISMA-ScR (Preferred Reporting Items for Systematic Reviews and Meta-Analyses extension for Scoping Reviews) guidelines [40,41]. Before initiating the review, a review protocol was developed, stored in the institutional database, and followed. It was not published publicly. Second, the identified barriers were systematically mapped with respect to the health profession, the context of care, and the research method.

### Eligibility Criteria

Only articles that have been published since June 2017 were eligible to ensure that the studies applied technology that is comparable with current standards. Publications that met the inclusion criteria were eligible (Textboxes 1 and 2).

**Inclusion criteria.**

**Article type**

- Original studies and journal articles

**Language**

- English or German

**Article scope**

- Article reports barriers or challenges with video call–based telehealth interventions

**Health professions**

- Barriers or challenges are based on the perspective of physical therapists, occupational therapists, speech and language therapists or audiologists, orthoptists, dieticians, nurses, or midwives

**Methods**

- Survey, interview, or focus group results

**Telehealth target**

- Video call–based telehealth targeted patient-to-provider interaction

**Time**

- Published since June 2017

**Exclusion criteria.**

**Article type**

- Review articles, conference papers, abstracts, editorials, newspaper articles, study protocols, and other formats

**Language**

- Other languages

**Article scope**

- Article reports no barriers or challenges and article reports barriers or challenges with other telehealth interventions than video call based (if other technologies were included in the study, the results must be clearly distinguishable)

**Health professions**

- Barriers or challenges are based on the perspective of patients, caregivers, medical doctors, or other health professionals. If they were included in the study, the results must be clearly distinguishable

**Methods**

- Results from other resources

**Telehealth target**

- Video call–based telehealth targeted academic education or continuing education and video call–based telehealth targeted provider-to-provider interaction

**Time**

- Published before June 2017

### Search and Selection Strategy

A comprehensive literature search was carried out in the PubMed and CINAHL databases on June 22, 2022, and updated on January 3, 2023. In addition, citation tracking was performed to identify additional relevant literature. Relevant topics for the search were video call–based telehealth; barriers or challenges; health professions of interest; and resources from interviews, focus groups, surveys, or questionnaires. The complete search strings can be found in Multimedia Appendix 1.

All references were imported to Zotero (Corporation for Digital Scholarship). In a first step, all duplicates were eliminated, and then all titles and abstracts of the sources were screened for inclusion criteria. The full text of the remaining articles was obtained, thoroughly reviewed for eligibility, and searched for further publications that met the inclusion criteria by citation tracking.

### Data Charting Process

The full-text articles of the selected publications were imported into MAXQDA (2022; VERBI GmbH), a software for qualitative content analysis. Each publication was coded for country, health profession, and target group of telehealth intervention to compare basic characteristics. The sample sizes were noted. Furthermore, all articles were thoroughly examined for the mentioned barriers, and codes were inductively assigned to text passages. The inductive coding process involved identifying patterns, themes, and categories in the data without the use of preexisting codes to allow for the identification of new and unexpected themes that may not have been anticipated. The codes were continuously refined, structured, and consolidated.

Finally, every publication had a code for country, health profession, target group, and if applicable, the following barrier codes were assigned: interpersonal issues, administration issues, practice issues, patient issues, environmental issues, telehealth attributes, technology issues, policy and regulation issues, and others. Subcodes for topics requiring further refinement were created.

### Synthesis of Results

Individual coding (passages with an assigned code) was analyzed and summarized for each code topic narratively. Similarities in the barriers mentioned were identified, and barriers for specific health professions or target groups were determined. Each barrier mentioned was equal in weight. A quantitative outline of barriers was performed only with respect to the number of articles that mentioned each barrier but not in terms of how often it was mentioned within an article.

## Results

### Overview

**Figure 1:**
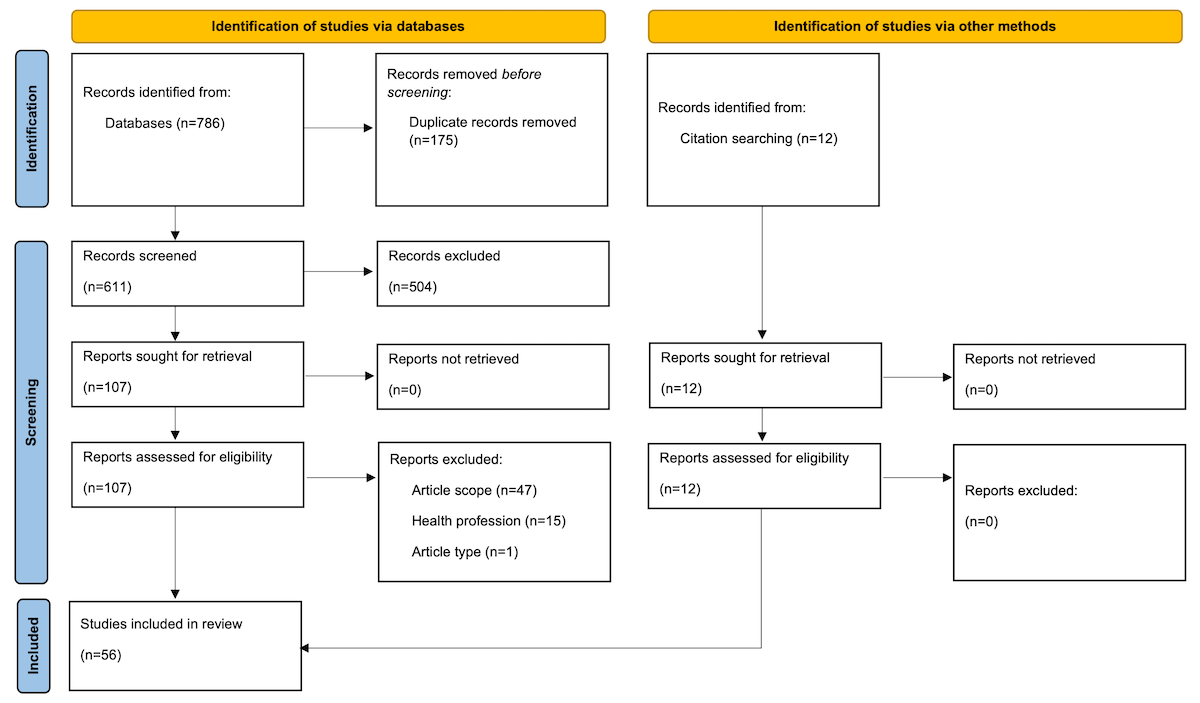
Study selection process.

The search yielded 786 records (n=609 in June 2022 and n=177 in January 2023). A total of 611 records were screened (n=474 in June 2022 and n=137 in January 2023), and 107 full-text papers were assessed for eligibility (n=77 in June 2022 and n=30 in January 2023). Finally, the review included 56 publications (n=44 in June 2022 and n=12 in January 2023). The selection process is illustrated in the flowchart (Figure 1).

### Study Characteristics

Table 1 presents all included publications with details for authors, publication year, country, included professions, health context, and assessment methods.

The included studies were published in 2017 (n=1) [42], 2018 (n=2) [43,44], 2019 (n=2) [45,46], 2020 (n=2) [47,48], 2021 (n=23) [49-71], and 2022 (n=26) [29,72-96]. Of the 56 papers, 48 (86%) papers focused on COVID-19 [29,47,49,51-56,58-96]. In total, 80% (45/56) of publications focused on 1 specific health care profession [29,42-47,49-51,53-59,63-66,68-75,77-81,83-86,88-91,93,94,96], and 20% (11/56) of articles targeted a mix of health care professions [48,52,60-62,67,76,82,87,92,95]. Most articles covered barriers for PTs (29/56, 52%) [43,48,49,51-53,55-57,59,61-65,67,74,76-82,84,85,87,92,95], followed by those for SLTs and audiologists (18/56, 32%) [29,48,50,52,59-61,73,79-81,83,85,92-94,96]. Furthermore, publications were concentrated on OTs (15/56, 27%) [45,47,52,60-62,67,72,73,76,82,87,92,95,96], nurses (5/56, 9%) [42,44,69,86,88], dieticians (4/56, 7%) [48,58,68,70], and midwives (2/56, 4%) [46,60]. No studies with orthoptists were included.

**Figure 2:**
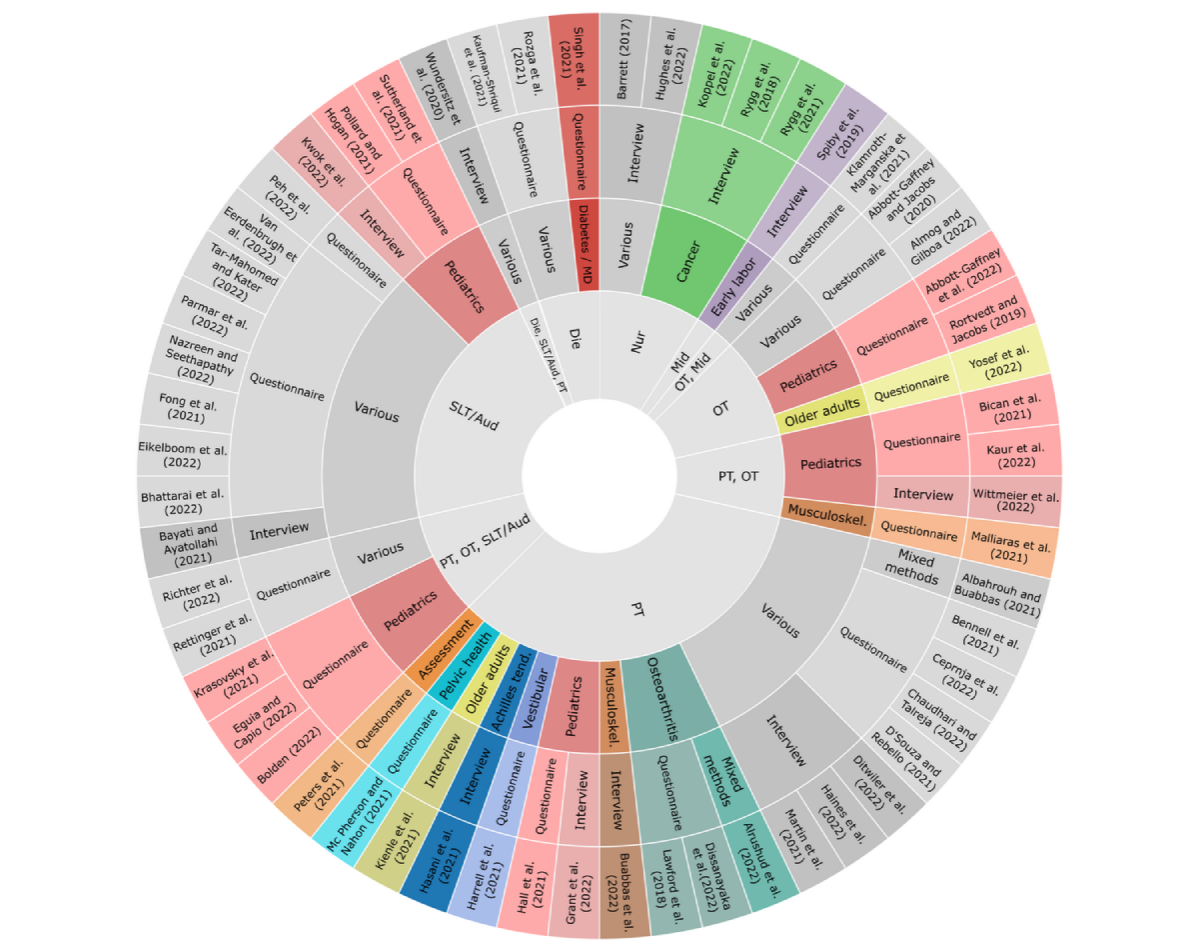
Mapping of study characteristics. Summary of the study characteristics from inner to outer circle: health profession, health context, method, authors, and year. Aud: audiologist; Die: dietitian; Mid: midwife; Nur: nurse; OT: occupational therapist; PT: physical therapist; SLT: speech and language therapist.

The studies focused on various health contexts. Health professionals treated various target groups (27/56, 48%) [42,45,47-51,53,54,58,60,63,67,73,75,78,79,81,83,85,86,89-94] or focused on specific target groups, such as children (13/56, 23%) [29,45,52,55,61,66,71,72,76,82,84,87,95] or older adults (2/56, 4%) [59,96], or patients with specific diseases or issues, such as musculoskeletal disorders (2/56, 4%) [62,77], cancer (3/56, 5%) [44,69,88], osteoarthritis (3/56, 5%) [43,74,80], vestibular disorders (1/56, 2%) [56], early labor (1/56, 2%) [46], diabetes (1/56, 2%) [70], pelvic health (1/56, 2%) [64], and Achilles tendinopathy (1/56, 2%) [57]. Overall, 2% (1/56) of studies focused on the execution of assessments via telehealth [65]. Figure 2 shows the study characteristics based on the included professions and health contexts [29,42-96].

**Figure 3:**
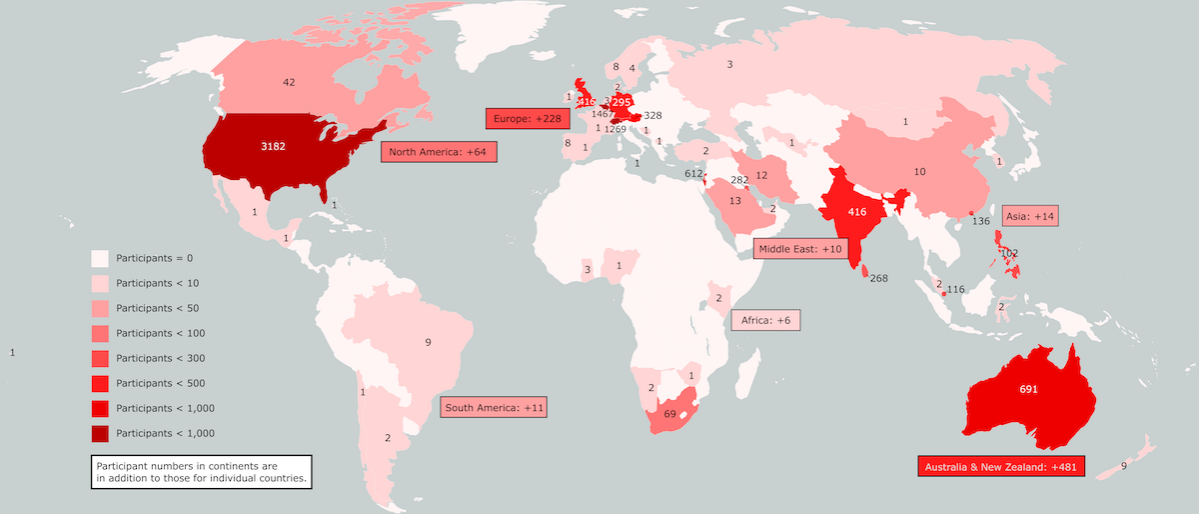
Total number of participants in the included studies according to region. The combined participant numbers of the included studies were based on the country, continent, or region.

The publications were from the United States (12/56, 21%) [45,47,52,55,56,68,70,72,76,81,87,88], Australia (12/56, 21%) [43,48,51,57,62-64,71,78,83-85], the United Kingdom (5/56, 9%) [42,46,66,86,90], Israel (4/56, 7%) [58,61,73,96], India (4/56, 7%) [53,75,79,89], Canada (3/56, 5%) [29,65,95], Germany (2/56, 4%) [59,92], Kuwait (2/56, 4%) [49,77], Norway (2/56, 4%) [44,69], Iran (1/56, 2%) [50], Hong Kong (1/56, 2%) [54], Switzerland (1/56, 2%) [60], Austria (1/56, 2%) [67], Belgium (1/56, 2%) [94], Singapore (1/56, 2%) [91], South Africa (1/56, 2%) [93], the Philippines (1/56, 2%) [82], Sri Lanka (1/56, 2%) [80], and Saudi Arabia (1/56, 2%) [74]. Study participants from a variety of continents and countries were included: 27% (15/56) of the participants were from North America, 21% (12/56) were from Europe and Australia each, 14% (8/56) were from the Middle East and South and East Asia each, and 2% (1/56) were from Africa. A visual representation of the worldwide distribution of study participants can be found in Figure 3.

Questionnaires (37/56, 66%) were used with a total of 10,245 health professionals [43,45,47,51-56,58,60-62,64-68,70-73,75,76,78-80,82,83,87,89-94,96], and interviews or focus groups (17/56, 30%) were conducted with 288 health professionals [29,42,44,46,48,50,57,59,63,69,77,81,84-86,88,95]. The methods used were mixed in 2 publications [49,74]. The evaluation methods, according to the included professions and their combined sample sizes, are presented in Table 2.

**Table 1:** Characteristics of sources.

Study	Country	Professions	Health context^a^	Methods^b^
Abbott-Gaffney and Jacobs [47], 2020	United States	OT^c^	Various	Questionnaire
Abbott-Gaffney et al [72], 2022	United States	OT	Pediatrics	Questionnaire
Albahrouh and Buabbas [49], 2021	Kuwait	PT^d^	Various	Mixed methods
Almog and Gilboa [73], 2022	Israel	OT	Various	Questionnaire
Alrushud et al [74], 2022	Saudi Arabia	PT	Osteoarthritis	Mixed methods
Barrett [42], 2017	United Kingdom	Nur^e^	Various	Interview
Bayati and Ayatollahi [50], 2021	Iran	SLT^f^ and Aud^g,h^	Various	Interview
Bennell et al [51], 2021	Australia	PT	Various	Questionnaire
Bhattarai et al [75], 2022	India	SLT and Aud	Various	Questionnaire
Bican et al [52], 2021	United States	PT and OT	Pediatrics	Questionnaire
Bolden [76], 2022	United States	PT, OT, and SLT and Aud	Pediatrics	Questionnaire
Buabbas et al [77], 2022	Kuwait	PT	Musculoskeletal	Interview
Ceprnja et al [78], 2022	Australia	PT	Various	Questionnaire
Chaudhari and Talreja [79], 2022	India	PT	Various	Questionnaire
Dissanayaka et al [80], 2022	Sri Lanka	PT	Osteoarthritis	Questionnaire
Ditwiler et al [81], 2022	United States	PT	Various	Interview
D’Souza and Rebello [53], 2021	India	PT	Various	Questionnaire
Eguia and Capio [82], 2022	Philippines	PT, OT, and SLT and Aud	Pediatrics	Questionnaire
Eikelboom et al [83], 2022	Australia	SLT and Aud	Various	Questionnaire
Fong et al [54], 2021	Hong Kong	SLT and Aud	Various	Questionnaire
Grant et al [84], 2022	Australia	PT	Pediatrics	Interview
Haines et al [85], 2022	Australia	PT	Various	Interview
Hall et al [55], 2021	United States	PT	Pediatrics	Questionnaire
Harrell et al [56], 2021	United States	PT	Vestibular	Questionnaire
Hasani et al [57], 2021	Australia	PT	Achilles tendinopathy	Interview
Hughes et al [86], 2022	United Kingdom	Nur	Various	Interview
Kaufman-Shriqui et al [58], 2021	Israel	Die^i^	Various	Questionnaire
Kaur et al [87], 2022	United States	PT and OT	Pediatrics	Questionnaire
Kienle et al [59], 2021	Germany	PT	Older adults	Interview
Klamroth-Marganska et al [60], 2021	Switzerland	OT and Mid^j^	Various	Questionnaire
Koppel et al [88], 2022	United States	Nur	Cancer	Interview
Krasovsky et al [61], 2021	Israel	PT, OT, and SLT and Aud	Pediatrics	Questionnaire
Kwok et al [29], 2022	Canada	SLT and Aud	Pediatrics	Interview
Lawford et al [43], 2018	Australia	PT	Osteoarthritis	Questionnaire
Malliaras et al [62], 2021	Australia	PT and OT	Musculoskeletal	Questionnaire
Martin et al [63], 2021	Australia	PT	Various	Interview
McPherson and Nahon [64], 2021	Australia	PT	Pelvic health	Questionnaire
Nazreen and Seethapathy [89], 2022	India	SLT and Aud	Various	Questionnaire
Parmar et al [90], 2022	United Kingdom	SLT and Aud	Various	Questionnaire
Peh et al [91], 2022	Singapore	SLT and Aud	Various	Questionnaire
Peters et al [65], 2021	Canada	PT	Assessment	Questionnaire
Pollard and Hogan [66], 2021	United Kingdom	SLT and Aud	Pediatrics	Questionnaire
Rettinger et al [67], 2021	Austria	PT, OT, and SLT and Aud	Various	Questionnaire
Richter et al [92], 2022	Germany	PT, OT, and SLT and Aud	Various	Questionnaire
Rortvedt and Jacobs [45], 2019	United States	OT	Pediatrics	Questionnaire
Rozga et al [68], 2021	United States	Die	Various	Questionnaire
Rygg et al [44], 2018	Norway	Nur	Cancer	Interview
Rygg et al [69], 2021	Norway	Nur	Cancer	Interview
Singh et al [70], 2021	United States	Die	Diabetes or metabolic disorder	Questionnaire
Spiby et al [46], 2019	United Kingdom	Mid	Early labor	Interview
Sutherland et al [71], 2021	Australia	SLT and Aud	Pediatrics	Questionnaire
Tar-Mahomed and Kater [93], 2022	South Africa	SLT and Aud	Various	Questionnaire
Van Eerdenbrugh et al [94], 2022	Belgium	SLT and Aud	Various	Questionnaire
Wittmeier et al [95], 2022	Canada	PT and OT	Pediatrics	Interview
Wundersitz et al [48], 2020	Australia	Die, SLT and Aud, and PT	Various	Interview
Yosef et al [96], 2022	Israel	OT	Older adults	Questionnaire

^a^Health context refers to specific patient or target groups or matter of interest in the included study.

^b^Methods were summarized as “Interview” when individual interviews or focus groups were conducted, “Questionnaire” when participants gave written answers, and “Mixed methods” when both methods were applied.

^c^OT: occupational therapist.

^d^PT: physical therapist.

^e^Nur: nurse.

^f^SLT: speech and language therapist.

^g^Aud: audiologist.

^h^SLT and Aud were combined, as they are a single profession in some countries and 2 professions in others.

^i^Die: dietitian.

^j^Mid: midwife.

**Table 2:** Participants in the included studies according to profession and evaluation methods.

	OT^a^ (n=1519), n (%)	PT^b^ (n=2857), n (%)	SLT^c^ and Aud^d^ (n=2852), n (%)	Nur^e^ (n=68), n (%)	Die^f^ (n=2435), n (%)	Mid^g^ (n=679), n (%)	PT or OT (n=16), n (%)	PT, OT, or SLT and Aud (n=102), n (%)	Total (n=10,533), n (%)
Questionnaire	1515 (99.7)	2722 (95.3)	2822 (98.9)	—^h^	2433 (99.9)	630 (92.8)	16 (100)	102 (100)	10,245 (97.3)
Interview	4 (0.3)	135 (4.7)	30 (1.1)	68 (100)	2 (0.1)	49 (7.2)	—	—	288 (2.7)

^a^OT: occupational therapist.

^b^PT: physical therapist.

^c^SLT: speech and language therapist.

^d^Aud: audiologist.

^e^Nur: nurse.

^f^Die: dietitian.

^g^Mid: midwife.

^h^Not available.

### Barriers to Video Call–Based Telehealth

**Figure 4:**
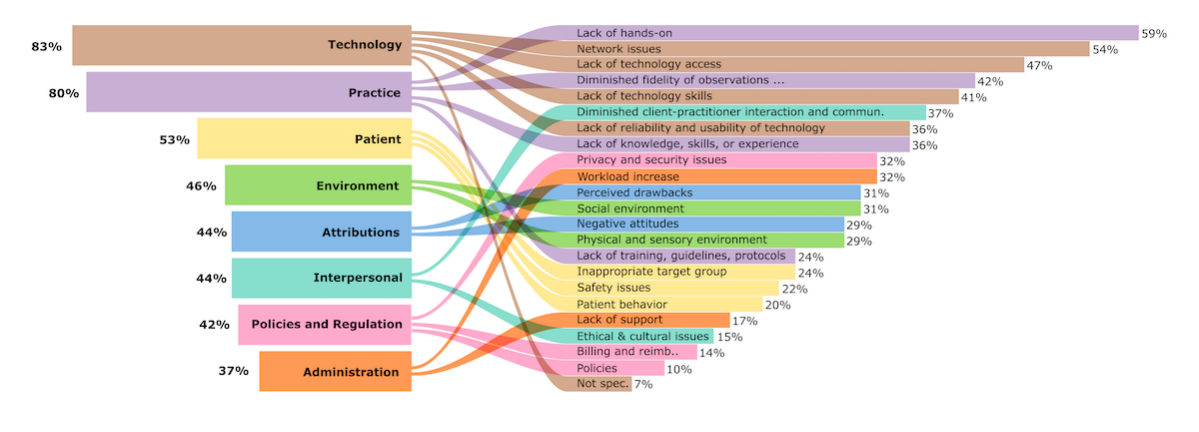
Barriers addressed by the publications according to category and subcategory in total. The bars on the left side represent the percentage of publications reporting barriers in the respective category. The bars on the right side represent the percentages of publications reporting barriers in the respective subcategory.

Barriers were assigned to 8 categories: technology issues, practice issues, patient issues, environmental issues, attributions, interpersonal issues, policies and regulations, and administration issues. Technology issues were found to encompass several subcategories, including limited access to technology, concerns about the reliability and usability of technology, network issues, a lack of technology skills among health care providers and patients, and other unspecified technological barriers. The analysis indicated that practice issues covered aspects such as diminished fidelity of observations and poor conditions for visual instructions; a lack of knowledge, skills, or experience; a lack of training, guidelines, or protocols; and the inability to provide hands-on care. Subcategories attributed to patient issues included addressing inappropriate target groups, managing patient behaviors, and addressing safety issues. Furthermore, physical, sensory, and social environmental issues were identified. Barriers to telehealth identified under the attributions category were negative attitudes toward telehealth from providers, patients, and caregivers, along with perceived drawbacks associated with telehealth use. Moreover, policy and regulation issues, which included privacy and security issues, billing and reimbursement topics, and workplace or health policies, were relevant barriers. Interpersonal barriers included reduced client-practitioner interaction and communication as well as the presence of ethical and cultural concerns. Finally, administration issues, such as a lack of support and a perceived increase in workload, were identified. A detailed description of the barriers for each subcategory and their references are provided in Table 3. The percentages of publications mentioning this barrier are depicted in Figure 4.

**Figure 5:**
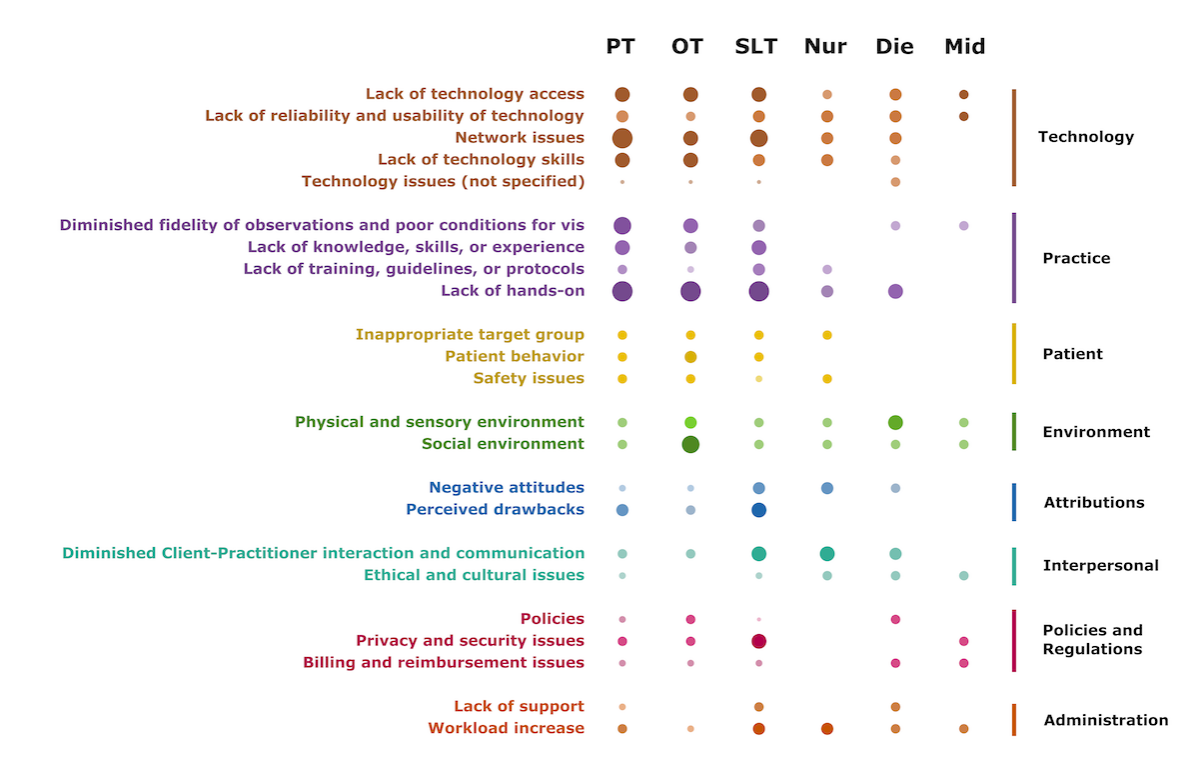
Barriers addressed by the publications according to the explored health profession. The size of the dots represents the relative number of documents addressing this barrier (row) based on this health profession (column). Die: dietitian; Mid: midwife; Nur: nurse; OT: occupational therapist; PT: physical therapist; SLT: speech and language therapist.

Publications reporting the barriers of PT, OT, and SLT covered all main categories, whereas nurse publications did not report any policy and regulation barriers. Dietitian and midwife publications had no information about patient issues, and midwife publications did not address category attributions. An overview of the distribution of reported barriers in the health profession is shown in Figure 5.

For further reference, Multimedia Appendix 2 provides an overview of the categories and subcategories of the barriers addressed in the individual studies [29,42-96].

**Table 3:** Details of each category and subcategory and the corresponding references.

Categories and subcategories			References
**Technology issues**			
	Lack of technology access	Availability of adequate equipment [45,46,49,54,55,59,62,64,68,70,71,73,77,78,83-87,90,92-95] Access of older adults [59,68] Patient groups in rural areas [55,84] Technology costs [45,49,53,54,62,64,71,72,89,93]	
	Lack of reliability and usability of technology	Insufficient quality of technology [62,70,94] Unreliability of technology [42,46,48,51,52,57,62,65-67,72,86,89,91] Lack of user-friendly software [49,53,59,70,95]	
	Network issues	Connection failure or limited bandwidth [44,47-49,51-53,55-57,59,62,64-66,70,71,76-79,84,85,87-90,93-95]	
	Lack of technology skills	Limited technology skills or confidence in use [51,59] of patients [44,49,51,56,57,59,62,64,67,70-74,79,83,86,90,95,96] of health professionals [29,62,70,72,81-84,90,95]	
	Technological barriers	Not specified [53,54,58,72]	
**Practice issues**			
	Diminished fidelity of observations and poor conditions for visual instructions	Observations or assessment options decreased [43,47,50,51,56,57,61-63,65,66,70,71,78,81,82,84,85,87,90,94,96] Fear to oversee red flags [49,59,62,78] Small screen size or pixilation [78] Video lag [65] Single-camera angle [56,57,62,66] Assessment that required walking [56] Lightning glare in glasses during eye exam [56] Sound quality [66] Loss of perception of smell [60] Lack of experience to “fill the blanks” [63] Lack of perception of the strengths of the patient [59,90] Lack of ability to read body language [70,90] Demonstration of exercises [59,62]	
	Lack of knowledge, skills, or experience	Patients lack knowledge about the role of technology in rehabilitation [49] Therapist skills [55] Lack of knowledge and experience [29,47,55,59,63,64,67,71,85,90,92,94,95] Lack of confidence [43,67,79,80,91] Lack of activity options for telehealth [47,64,72,91] Hard to engage clients [76] Missing evidence base [67,75,90]	
	Lack of training, guidelines, or protocols	Lack of appropriate telehealth training [49,53,54,64,76,83-85,90,92,95] Learning curve required [76,95] Missing guidelines or standard protocols [77,83,84,88,90,91]	
	Lack of hands-on methods	Lack of direct hands-on methods [42,43,45,47-51,53-56,58-62,64,66,67,70,73,76,77,80-84,88,90,92,94-96] Inability of physical examinations [50,51,58,61,62,64,66,70,81,84,88,94,95] Inability to demonstrate, intervene, or provide assistance with hands [47,48,51,62,73,76,81,82,84,92,94,95] Inability to program or adjust hearing aids remotely [83,90] Inability to perform swallowing assessments [48] Inability to take anthropometric measurements [70] Hands-on absence in terms of presence in a broader sense [42,88]	
**Patient issues**			
	Inappropriate target group	Not appropriate for all groups of patients [54,73,81,91,92] Age [55,73,74,85,90] Diagnosis [50,55,64,85,90] Vision and hearing abilities [73,74,88] Cognitive abilities [73,96] Language skills [85,88] Personal preference [81] Technology experience [88] Inappropriate referrals [47]	
	Patient behavior	Maintenance of patient attention [91,94] Especially with children [45,47,61,72,76,84] Children’s negative behavior increased [55] or harder to manage [66,91] Children’s engagement reduced [66,97] Inferior patient compliance [53,94]	
	Safety issues	Safety risks [43,62,64,67,73,80,81,94,96] High fall risk [51] Missing safeguard measures [56,62,73,86,96] Increased need for multitasking in telehealth could increase the risk of fall [59] Unsupervised exercise execution might lead to incorrect technique [51] Perception of safety risks might be harder [59,64]	
**Environmental issues**			
	Physical and sensory environment	Limit telehealth experience for patients and health care professionals [58,85] Noise distractions [61,62,70,73,85,93] Poor camera angles [48] Limited space [62,73,84,86,95] Lack of privacy [46,64,73,85,95] Lack of material needed [48,52,72,73,81]	
	Social environment	Absence of people to support with technology or other tasks [45,47,56,70,72,86,87,95] Missing language interpreters [46,52,70] Struggling, overwhelmed, or reluctant relatives [55,73,76,82,94,96]	
**Attributions**			
	Negative attitudes	Negative attitudes or reluctance of health care providers [49,50,53,86] Negative attitudes or reluctance of patients, clients, or caregivers [44,45,50,54,59,62,66,68,71,87,91,92,94] Causation of nocebo effects [59] Lack of understanding of telehealth [45,71] Fear of scams by especially older clients [68] Worsened public perception of the profession [83] Fear of job automatization [90]	
	Perceived drawbacks	Quality concerns [87,89] Lack of perceived clinical usefulness [43,49,53] Lack of clinical effectiveness [43,49,50,54,62,64,66,67,77,78,80,84,91,92,94] Slower progress of treatment [78] Reduced patient satisfaction [67] Diminished capability to meet urgent cases [83]	
**Policy and regulation issues**			
	Privacy and security issues	Patient privacy at risk [43,45,46,49,50,59,72,80,84,95] Security, confidentiality, and data protection concerns [43,45,46,49,50,53,59,66,67,71,72,83,84,89-92,95]	
	Billing and reimbursement issues	Funding, reimbursement, and payment for telehealth services [46,62,64,67,70,72,79,91]	
	Policies	Workplace policies or missing workplace policies [70,72,77,95] Licensure issues when providing telehealth across state borders [47,72] Health policies [67]	
**Interpersonal issues**			
	Diminished client-practitioner interaction and communication	Limited interaction between the client and the practitioner [83,94] Harder to establish relationship or rapport [47,57,62,63,69,70,73,83-85,88,90,91,93] Harder to establish trust [50,73] Risk of impersonal care [54,67,83,90,93] Lack of human warmth [73] Impaired perception of client personality traits [59] Nuances get lost over the screen [73] Diminished communication quality in general [50,58,62,84,90,94] Language barriers [62,66,70,94] Increased possibility of misunderstandings [81,88,94] Risk of social isolation for patients [86] Risk of social isolation for providers [62,86]	
	Ethical and cultural issues	Web-based sessions of female providers in the presence of male caregivers [49] Cultural reasons for not accepting video calls [74] Discussion of private or embarrassing topics [83] Reacting to worrisome activities, seen in the home of patients [46] Examinations involving disrobing [64] Lack of comfort and support for difficult or emotional conversations [88] Ethical concerns, not specified [54] Spiritual or religious barriers, not specified [59,70]	
**Administration issues**			
	Lack of support	Lack of administrative and technical support [29,49,53,54,70,77,78,83,84,90] Inefficiencies in organization, scheduling, or visit preparation [70,78,90] Lack of knowledge of using the system or troubleshooting [29] Not enough referrals and promotions for telehealth [64]	
	Workload increase	Increased workload, pressure, or stress [49,53,55,66,82,86] Increased time need to organize and use telehealth [29,44,46,62,70,78,82,84,89-91,94,95] Increased level of fatigue [66,84-86,91,95] Worsening of appointment-related travel [83] Change in working hours [90]	

## Discussion

### Principal Findings

This review aimed to map and condense the perceived barriers of allied health professionals concerning the use of video call–based telehealth, as understanding these barriers is crucial for improving telehealth adoption, efficiency, and effectiveness. By providing information to health care professionals and health services on barrier identification, this study serves as the first step toward addressing and overcoming these obstacles. We included 56 publications that reported barriers for OTs, PTs, SLTs and audiologists, dieticians, midwives, and nurses. Barriers were assigned to 8 topics: technology issues, practice issues, patient issues, environmental issues, attributions, policy and regulation issues, interpersonal issues, and administration issues. Previous publications on telemedicine barriers also used similar, but also different, classification schemes. The Pan American Health Organization refers to barriers by technological, organizational, human, and economic environments [39]. Almathami et al [98] described internal and external barriers, and Scott Kruse et al [99] differentiated between organizational barriers and patient barriers. However, similar barriers underpin these different classifications.

The barriers mentioned most frequently pertain to technological issues. Although video call technologies are widespread in a large part of the population, certain groups or individuals lack suitable hardware or network access. This could be the case not only for some older adults but also for patient groups living in rural areas or those who are socioeconomically disadvantaged. The limited access of patient groups should be considered when telehealth services are offered to avoid possible disadvantages. Furthermore, the risk of a digital divide extends beyond mere access to technology. It also encompasses a lack of knowledge of digital health or technology skills [100,101], as they were reported in numerous included studies of this and previous reviews [99]. To facilitate the acceptance and effective use of telehealth tools, hardware and software must be designed to accommodate users’ needs [98]. According to common frameworks, perceived usefulness and perceived ease of use affect the acceptance of a technology and are further influenced by experience [102]. To overcome these barriers, it is important to develop and use video call software that meets the specific needs of providers and patients and adheres to usability principles. In addition, health professionals, as well as patients, need to have the knowledge and skills to use technology. Thorough preparation of video visits or calling on e-helpers can facilitate successful implementation [51,86,95]. However, it is essential to recognize that network issues may still occur, potentially causing interruptions during video calls and affecting the overall telehealth experience. These were the second most cited barriers in this review, showing similarity to the review by Almathami et al [98], where this barrier held the top rank. To reduce network problems, it would be necessary to invest in and prioritize the development and maintenance of robust and reliable telecommunication infrastructure. This includes improving broadband connectivity in rural and underserved areas, which are often specifically addressed by telehealth services. In addition, providing telehealth users with guidelines for troubleshooting network-related problems could further enhance the overall experience and success of telehealth initiatives.

Second, most publications have reported barriers related to practice issues. Health care provision involves various physical, verbal, and observational measures. Providing care on the web on the screen involves a variety of challenges concerning this matter. Although physical therapy often involves whole body movements and hands-on techniques, dietitians are more based on conversation. Occupational therapy is often oriented toward daily activities, incorporating many different objects, and speech and language therapy is dependent on good sound quality. However, most practice-related barriers are applicable to all involved health care professions. First, hands-on methods cannot be applied to examinations, demonstrations, interventions, and assistance. This barrier was mentioned in 59% (33/56) of the publications and therefore seems to represent a core barrier for the explored health professions. Notably, it was not represented as a key barrier in earlier reviews that included a wider range of health professions [98,99] and might be more specific for the professions examined in this study. To address the lack of hands-on methods, they must be replaced by other actions that target the same outcome of comparable quality. For this, health professionals need specific training, guidelines, or protocols that expand their skill sets. However, if there are no adequate replacements for hands-on techniques, telehealth services may solely not be appropriate. Practitioners could combine telehealth and in-person services or work closely with local health care professionals to coordinate in-person care when needed. This collaborative approach ensures that patients receive comprehensive care that combines the convenience of telehealth with the benefits of in-person care. Second, limitations are also prevalent with respect to the diminished conditions for observations. Issues that negatively contribute to this are poor video and audio quality, difficulties with camera placement, and a lack of perception of body language, as described previously [98]. Establishing a professional setting on both sides can help minimize this barrier. However, these might be associated with additional costs, which were also found to hinder telehealth adoption [99]. Furthermore, encouraging the development and implementation of digital assessment tools tailored for telehealth sessions could aid health care professionals in making more accurate observations despite the limitations of the web-based setting [103,104]. Training, guidelines, or protocols are needed to guide health professionals in creating optimal conditions for valid observations and high-quality therapy.

Telehealth provision, in addition to visual observations, is highly dependent on communication. Therefore, a limited interaction between the client and the practitioner was described as a barrier. There exists the fear that information might be lost along the way, and misunderstandings can occur. Moreover, it was mentioned that it is more difficult to establish relationships or trust. Such communication issues and perceptions of impersonal care have been reported previously [98,99]. Therefore, special emphasis should be placed on expanding the communication skills of health care providers to overcome those barriers [105-107]. Health care professionals should be trained to be more aware of and skilled in nonverbal communication cues, such as facial expressions, gestures, and body language. This can help to better understand patients’ needs, emotions, and reactions during telehealth sessions, thereby improving overall communication and rapport building. Moreover, health care professionals reported a decrease in communication quality, especially when language barriers were present. By implementing appropriate strategies, telehealth on the other hand could also help overcome language barriers when providers on the web collaborate with professional interpreters [108] or integrate language support features, such as real-time translation [109]. Comprehensive training modules that familiarize health care providers with the available language support services and their use in telehealth settings would be needed.

When discussing patient selection for telehealth services, it is crucial to consider several factors, including the demographic, physiological, and personal characteristics of patients. Some health professionals expressed the need for algorithms to establish clear inclusion and exclusion criteria. Certain diagnoses or health restrictions may limit a patient’s ability to interact effectively through telehealth or pose risks that cannot be adequately managed within this service model. Ensuring patient safety and addressing potential risks should be integral to the planning and management of telehealth services [110]. In the context of telehealth for children, some publications highlighted challenges related to maintaining attention, engagement, and managing challenging behaviors. To address these issues, health professionals may need to use innovative approaches in adapting methods and materials to suit web-based settings [111-113]. In addition, prioritizing coaching and support for relatives can help overcome barriers associated with telehealth in children [100,101].

Caregivers can play an important role in supporting the patient with technology or other tasks. Furthermore, they are directly addressed by health professions for training or support [114]. If they are not available or struggle with the responsibilities assigned, telehealth provision can be challenging, especially if patients depend on them [98]. Therefore, the social system of patients should be considered to determine whether telehealth services are appropriate. Furthermore, it is important to evaluate the physical and sensory environments of patients, as described by Almathami et al [98]. Depending on the goals and types of health care provision, space and material needs should be considered and evaluated. If possible, providers could conduct home assessments to evaluate the physical and sensory environment of patients and make recommendations for modifications or improvements to the space. In addition, a lack of privacy at home can be an issue. To address this barrier, health care providers can offer guidance on how to create a private space for telehealth visits, such as using a separate room or wearing headphones to ensure confidentiality. Finally, health care providers should consider alternative methods of care for patients who do not have access to a private space for telehealth visits.

Privacy issues also refer to the transmission of sensible health data over the internet and have been widely discussed [98,99]. Many health professionals mentioned that they feared that their patient’s privacy was at risk and that they had concerns about data protection. Telehealth requires secure platforms where data are encrypted to protect patient privacy and confidentiality [115]. Therefore, it is important to educate health care professionals about suitable platforms and provide guidelines so that they can make informed decisions about IT security measures [116]. Commercial platforms are not always in compliance with the General Data Protection Regulations [115] or the Health Insurance Portability and Accountability Act [98] and should be transparent about how they store, transmit, encrypt, and use data so that health providers can compare those measures with current guidelines [117].

In addition to fear of a lack of data privacy, other negative attitudes toward telehealth influence the acceptance of telehealth. This applies to the reluctance to telehealth of health care providers, patients, and caregivers, also described as resistance to change [99]. Most often, statements about clinical effectiveness or clinical usefulness were reported as reasons. The body of evidence for telehealth effectiveness varies depending on the health context and the involved health care professions. To give some examples, 2 meta-analyses found positive clinical results, even comparable with conventional face-to-face rehabilitation approaches, for PT and OT telerehabilitation services [118,119]. A systematic review of telepractice for adult speech language therapy supports the use of telepractice as an appropriate service delivery model [120]. Moreover, the benefits of using telepractice to provide parent-implemented interventions to children with autism spectrum disorders have been described [121]. Telehealth services from dieticians or nutritionists can improve protein intake and quality of life in malnourished older adults [122]. Yet, there is a lack of high-quality evidence and training courses to inform health care providers about current evidence.

Finally, all the mentioned barriers can lead to an increase in the workload and stress of health care professionals, especially when experience with telehealth has not been established. In such cases, providers may face challenges such as technological difficulties, ineffective communication with patients, and patient selection issues, which can lead to increased stress and a decrease in work efficiency. They need administrative and technological support, protocols, and guidelines to help them establish a well-functioning telehealth practice. To achieve sustainable telehealth services, it is important to ensure that health care professionals are willing and able to use these tools effectively. This means that health organizations must provide adequate resources and training to support the adoption and use of telehealth technology, as their acceptance is crucial to the success of sustainable telehealth services [123].

### Limitations

This review only covered original studies, which were found in the PubMed and CINAHL databases, in German or English language and were published since June 2017. Although these are widely recognized and commonly used databases in the field of health care research, this may have resulted in the exclusion of relevant studies published in other databases. However, efforts have been made to minimize this limitation using comprehensive search strings and citation tracking to identify additional relevant literature. Future studies could expand the search to include a wider range of databases to increase the comprehensiveness of the search strategy. Furthermore, only 1 researcher conducted the search, selection, and mapping process. A broader search process, with more languages and researchers included, might have increased the number of publications and reliability of the results.

Publications were selected based on included health care professions. In case, professions out of scope were involved, the author selected only information from health care professionals that were within the scope of all conscience but with the risk of interference. Furthermore, there were big differences between the number of publications and included participants in the health care profession of interest. Although more than half of the publications addressed barriers of PTs and another third addressed those of SLTs and audiologists, other professions were less represented. Furthermore, SLTs and audiologists were combined, as they are 1 profession in some countries, whereas in others, they are 2 separate professions. Owing to these quantitative differences, it is difficult to compare the mentioned barriers between these professions and discuss whether there are significant differences. Furthermore, the number of publications that mentioned a barrier might not reflect its relevance. Therefore, all numbers and corresponding figures should be interpreted with caution.

Another limitation is that this review focused on health contexts rather than health settings. The included studies covered a range of health professions and contexts of care but did not distinguish between different settings, such as acute, tertiary, or community settings. There may be differences in the barriers and challenges faced by telehealth interventions across different health settings; therefore, the findings of this study may not be generalizable to all health settings.

This review has a slightly more balanced distribution of publication sites when compared with other reviews that focused on telehealth barriers. It incorporates approximately half of the publications from North America (15/56, 27%) and Europe (12/56, 21%) and the other half from Australia (12/56, 21%), the Middle East (8/56, 14%), South and East Asia (8/56, 14%), and Africa (1/56, 2%). Earlier reviews that focused on telemedicine barriers reported nearly 73% [99] or even 84% [98] of references from North America or Europe. However, there is a huge underrepresentation of African countries especially.

This review covered most studies that were conducted during the COVID-19 pandemic. As this period was highly influenced by contact restrictions, many publications have included participants who used telehealth services as a matter of imperative necessity. This could have influenced their attitudes and may have led to increased confrontation with barriers that could have been prevented with more time for preparation. In addition, the review included only a limited number of prepandemic studies, which may limit the generalizability of our findings beyond the pandemic period. Nonetheless, no differences were observed in the reported barrier categories between studies with and without a COVID-19 context. However, further research is needed to examine the challenges and barriers to telehealth interventions in different settings and contexts.

Finally, qualitative and quantitative results from these studies were synthesized in this review. It should be considered that participants give different answers when they reply to open-ended or closed-ended questions.

### Conclusions

This study systematically reports the barriers of allied health professionals toward video call–based telehealth within 59 original studies with OTs, PTs, SLTs and audiologists, dieticians, midwives, and nurses. Through the review, a range of barriers were identified, including technology, practice, patient, environmental, attributions, policy and regulation, interpersonal, and administration issues. The results emphasize the need for stable infrastructure, education, training, guidelines, policies, and support systems to establish high-quality allied telehealth services. The identification of these barriers is crucial, as it highlights the areas where improvements are needed in telehealth services to better meet the needs of allied health professionals, nurses, and their patients. The information gathered in this review can be used to improve telehealth services in health care organizations.

## Appendix

Multimedia Appendix 1 Search strings used in PubMed and CINAHL databases.

Multimedia Appendix 2 Barriers addressed by the publications according to category and subcategory for each publication.

Multimedia Appendix 3 Full data excel sheet.

## Abbreviations

OT: occupational therapist
PRISMA-ScR: Preferred Reporting Items for Systematic Reviews and Meta-Analyses extension for Scoping Reviews
PT: physical therapist
SLT: speech and language therapist

## Data Availability

The data set analyzed during this study is available in Multimedia Appendix 3.
