# Supplementary material for: Barriers to Video Call–Based Telehealth in Allied Health Professions and Nursing: Scoping Review and Mapping Process
Source: J Med Internet Res. 2023 Aug 1;25:e46715. doi: 10.2196/46715 (PMC10427933; doi:10.2196/46715)
Supplement: Multimedia Appendix 1 [file jmir_v25i1e46715_app1.pdf]

Pubmed on *2022 June 22*

(teleconsultation [tiab] OR "tele-consultation" [tiab] OR telehealth [tiab] OR "tele-health" [tiab] OR teleintervention [tiab] OR "tele-intervention" [tiab] OR telemedicine [tiab] OR "tele-medicine" [tiab] OR telerehabilitation [tiab] OR "tele-rehabilitation" [tiab] OR teletherapy [tiab] OR "tele-therapy" [tiab] OR telepractice [tiab] OR "tele-practice" [tiab] OR telecare [tiab] OR "tele-care"[tiab] OR "online-therapy" [tiab] OR teleconf* OR video-call* OR video-conf*) AND (barrier* [tiab] OR challenge*[tiab]) AND (Dieti* [tiab] OR nutritionist* [tiab] OR audiolog* [tiab] OR nurs* [tiab] OR midwi* [tiab] OR orthopt* [tiab] OR "physical therap*" [tiab] OR "physiotherap*"[tiab] OR "occupational therap*" [tiab] OR "speech therap*" [tiab] OR "language therap*" [tiab] OR "speech and language therapist*" [tiab] OR "allied health professionals"[tiab]) AND (interview* OR "focus group*" OR survey* OR questionnaire*)

Filter: last 5 years

Cinahl on *2022 June 22*

(teleconsultation OR "tele-consultation" OR telehealth OR "tele-health" OR teleintervention OR "tele-intervention" OR telemedicine OR "tele-medicine" OR telerehabilitation OR "tele-rehabilitation" OR teletherapy OR "tele-therapy" OR telepractice OR "tele-practice" OR telecare OR "tele-care" OR "online-therapy" OR teleconf* OR video-call* OR video-conf*) AND (barrier* OR challenge*) AND (Dieti* OR nutritionist* OR audiolog* OR nurs* OR midwi* OR orthopt* OR "physical therap*" OR "physiotherap*" OR "occupational therap*" OR "speech therap*" OR "language therap*" OR "speech and language therapist*" OR "allied health professionals") AND (interview* OR "focus group*" OR survey* OR questionnaire*)

Filter: 06/2017-06-2022

Pubmed on 2023 January 03

(teleconsultation [tiab] OR "tele-consultation" [tiab] OR telehealth [tiab] OR "tele-health" [tiab] OR teleintervention [tiab] OR "tele-intervention" [tiab] OR telemedicine [tiab] OR "tele-medicine" [tiab] OR telerehabilitation [tiab] OR "tele-rehabilitation" [tiab] OR teletherapy [tiab] OR "tele-therapy" [tiab] OR telepractice [tiab] OR "tele-practice" [tiab] OR telecare [tiab] OR "tele-care"[tiab] OR "online-therapy" [tiab] OR teleconf* OR video-call* OR video-conf*) AND (barrier* [tiab] OR challenge*[tiab]) AND (Dieti* [tiab] OR nutritionist* [tiab] OR audiolog* [tiab] OR nurs* [tiab] OR midwi* [tiab] OR orthopt* [tiab] OR "physical therap*" [tiab] OR "physiotherap*"[tiab] OR "occupational therap*" [tiab] OR "speech therap*" [tiab] OR "language therap*" [tiab] OR "speech and language therapist*" [tiab] OR "allied health professionals"[tiab]) AND (interview* OR "focus group*" OR survey* OR questionnaire*)

Filter: since 2022 June 22

Cinahl on *2023 January 03*

(teleconsultation OR "tele-consultation" OR telehealth OR "tele-health" OR teleintervention OR "tele-intervention" OR telemedicine OR "tele-medicine" OR telerehabilitation OR "tele-rehabilitation" OR teletherapy OR "tele-therapy" OR telepractice OR "tele-practice" OR telecare OR "tele-care" OR "online-therapy" OR teleconf* OR video-call* OR video-conf*) AND (barrier* OR challenge*) AND (Dieti* OR nutritionist* OR audiolog* OR nurs* OR midwi* OR orthopt* OR "physical therap*" OR "physiotherap*" OR "occupational therap*" OR "speech therap*" OR "language therap*" OR "speech and language therapist*" OR "allied health professionals") AND (interview* OR "focus group*" OR survey* OR questionnaire*)

Filter: since 2022 June 22
